# Hausdorff Dimension and Topological Entropies of a Solenoid

**DOI:** 10.3390/e22050506

**Published:** 2020-04-28

**Authors:** Andrzej Biś, Agnieszka Namiecińska

**Affiliations:** Faculty of Mathematics and Computer Science, Banacha 22, 90-235 Łódź, Poland; a.namiecinska@wp.pl

**Keywords:** entropy, Hausdorff measure, Hausdorff dimension, box dimension, solenoid, locally expanding map

## Abstract

The purpose of this paper is to elucidate the interrelations between three essentially different concepts: solenoids, topological entropy, and Hausdorff dimension. For this purpose, we describe the dynamics of a solenoid by topological entropy-like quantities and investigate the relations between them. For L-Lipschitz solenoids and locally λ—expanding solenoids, we show that the topological entropy and fractal dimensions are closely related. For a locally λ—expanding solenoid, we prove that its topological entropy is lower estimated by the Hausdorff dimension of *X* multiplied by the logarithm of λ.

## 1. Introduction

A solenoid, which was introduced to mathematics by Vietoris [1] as the topological object, can be presented either in an abstract way as an inverse limit or in a geometric way as a nested intersection of a sequence of tori. The classical construction of Vietoris was modified by McCord [2], Williams [3], and others. Since the publication of William’s paper on expanding attractors [3], inverse limit spaces have played a key role in dynamical systems and in continuum theory. Smale [4] introduced the solenoid to dynamical systems as a hyperbolic attractor.

In the paper, we investigate the complexity of a solenoid determined by the sequence $f_{\infty }={\left(f_{n}:X\to X\right)}_{n=1}^{\infty }$ of continuous epimorphisms of a compact metric space (X,d), called bonding maps. By *solenoid* determined by $f_{\infty }$, we mean the inverse limit

$$X_{\infty }=\lim_{⟵}\left(X,f_{k}\right):=\left\{{\left(x_{k}\right)}_{k=0}^{\infty }:x_{k-1}=f_{k}\left(x_{k}\right)\right\}.$$

As $X_{\infty }$ is uniquely determined by $f_{\infty }$, we will use these two terms interchangeable.

A solenoid is both a compact connected metric space (continuum) and a dynamical object of complicated structure. If additionally *X* is an abelian group then the compact metric space $X_{\infty }$ is an abelian group as well. In mathematical literature, one can also find a more restrictive definition of the solenoid as a finite-dimensional, connected, compact abelian group. These solenoids generalize torus groups, and their entropic properties have been studied by Berg [5], Lind and Ward [6], Einsiedler and Lindenstrauss [7], and others. A less restrictive definition of the solenoid was considered in [8,9,10].

Solenoids are a natural generalization of classical dynamical systems. In contrast with the classical dynamical systems, the properties of solenoid entropies have not been fully investigated. In the paper, we consider several different definitions of entropy-like quantities for a solenoid $f_{\infty }$: topological entropy $h_{top}\left(f_{\infty }\right)$, topological cover entropy $h_{top-cov}\left(f_{\infty }\right)$, and topological dimensional entropy $h_{top-dim}\left(f_{\infty }\right).$

Both nonautonomous dynamical systems and solenoids are determined by compositions of continuous self-maps; therefore, in both cases, the entropy-like quantities that capture complexities of dynamical systems can be similar. For example, the topological entropy of a solenoid coincides with the topological entropy of a nonautonomous dynamical system defined in [11]. In this paper, we derive the following relations between the entropies of a solenoid which were previously known for continuous maps on compact metric spaces, and we obtained the following results.

**Theorem** **1.** $h_{top-dim}\left(f_{\infty }\right)\leq h_{top-cov}\left(f_{\infty }\right).$

**Theorem** **2.** $h_{top}\left(f_{\infty }\right)=h_{top-cov}\left(f_{\infty }\right).$

In 2002, Milnor [12] stated two questions related to the classical dynamical system: “Is entropy of it effectively computable?” “Given an explicit dynamical system and given ϵ>0, is it possible to compute the entropy with maximal error of ϵ?” In most cases the answer is negative. For the recent results on computability of topological entropy, we recommend [13,14].

Therefore, in mathematical literature, there were many attempts to estimate entropy of dynamical systems by Lyapunov exponents, volume growth, Hausdorff dimension, or fractal dimensions.

The theory of Carathéodory structures, introduced by Pesin [15] for a single map, has been applied in [11] to get some estimations of the topological entropy of a nonautonomous dynamical system. To show a comprehensive picture and beauty of dynamics of solenoids, we rewrite the Theorem 3 in [11] to express complexity of so called L-Lipschitz solenoid. A solenoid $f_{\infty }={\left(f_{n}:X\to X\right)}_{n=1}^{\infty }$ is called L-Lipschitz if it consists of L-Lipschitz epimorphisms; the following inequality holds.

**Theorem** **3.** *Assume that $f_{\infty }={\left(f_{n}:X\to X\right)}_{n=1}^{\infty }$ is a L-Lipschitz solenoid with L>1. Then, for any Y⊂X, we obtain*

$$HD\left(Y\right)\geq \frac{h_{top-dim}\left(\left(f_{\infty }\right),Y\right)}{\log(L)},$$

*where HD(Y) is the Hausdorff dimension of Y.*

Finally, we investigate so called locally λ—expanding solenoids, in the sense of Ruelle [16] (see Definition 5). We prove that the topological entropy of such a solenoid, defined on the space X, is related to the upper box dimension of *X* multiplied by the logarithm of λ. We obtained the following inequalities.

**Theorem** **4.** *Given a locally λ—expanding solenoid $f_{\infty }={\left(f_{n}:X\to X\right)}_{n=1}^{\infty }$. Then,*

$$h_{top}\left(f_{\infty }\right)\geq \left(\log\lambda \right)\cdot \bar{\dim_{B}\left(X\right)}\geq \left(\log\lambda \right)\cdot HD\left(X\right),$$

*where $\bar{\dim_{B}\left(X\right)}$ is the upper box dimension of X.*

The paper is organized as follows. In Section 2, we introduce several definitions of entropy-like quantities for a solenoid: topological entropy, topological cover entropy, and topological dimensional entropy. In Section 3, we prove the relations between them (Theorems 1 and 2). Section 4 is devoted to L-Lipschitz solenoids; we present Theorem 3. Finally, in Section 5, we investigate locally λ—expanding solenoids and prove Theorem 4.

## 2. Topological Entropies of a Solenoid

In 1965, Adler, Konheim, and McAndrew [17] introduced a definition of topological entropy for the classical dynamical system (i.e., a pair (X,f), where *X* is a topological space and f:X→X is a continuous map) as a non-negative number assigned to an open cover of X. A different definition of entropy of a continuous self-map defined on a compact metric space was introduced by Bowen [18] and independently by Dinaburg [19]. In [20], Bowen proved that the definitions are equivalent. Nowadays, topological entropy is a main notion in topological dynamics. In the paper, we present a few generalizations of the classical topological entropy of a single map to solenoids.

In the paper, we consider a solenoid determined by a sequence $f_{\infty }={\left(f_{n}:X\to X\right)}_{n=1}^{\infty }$ of continuous epimorphisms of a compact metric spaces (X,d). Thus, we obtain that the solenoid is a generalized dynamical system. Its complexity, complicated topological structure, and chaos can be measured by several entropy-like quantities. First, we introduce topological entropy via (n,ϵ)—separated sets.

### 2.1. Topological Entropy of a Solenoid via (n,ϵ)—Separated or (n,ϵ)—Spanning Sets

Let B(x,r)={y∈X:d(x,y)≤r} denote a closed ball in the metric space (X,d) centered at x∈X and with radius r.

**Definition** **1.** *Fix ϵ>0, n∈N. A subset F⊂X is called (n,ϵ)-spanning if for any x∈X there exists y∈F such that*

$$\max\{d\left(f_{i}\circ f_{i+1}\circ \ldots \circ f_{n}\left(x\right),f_{i}\circ f_{i+1}\circ \ldots \circ f_{n}\left(y\right)\right):i\in \left\{1,\ldots ,n\right\}\}\leq \epsilon .$$

*Let r(n,ϵ):=min{card(F):Fis(n,ϵ)-spanningset}.*

*A set E⊂X is called (n,ϵ)-separated if for any pair of distinct points x,y∈E we have*

$$\max\{d\left(f_{i}\circ f_{i+1}\circ \ldots \circ f_{n}\left(x\right),f_{i}\circ f_{i+1}\circ \ldots \circ f_{n}\left(y\right)\right):i\in \left\{1,\ldots ,n\right\}\}>\epsilon .$$

*Let s(n,ϵ):=max{card(E):Eis(n,ϵ)-separated}.*

The following two lemmas are a reformulation of Definition 1.

**Lemma** **1.** *A set F⊂X is (n,ϵ)—spanning if and only if*

$$X=\underset{y\in F}{⋃}\overset{n}{\underset{i=1}{⋂}}{\left(f_{i}\circ f_{i+1}\circ \ldots \circ f_{n}\right)}^{-1}B\left[\left(f_{i}\circ f_{i+1}\circ \ldots \circ f_{n}\right)\left(y\right),\epsilon \right].$$

**Lemma** **2.** *A set E⊂X is (n,ϵ)-separated if and only if for any x∈E the set ${⋂}_{i=1}^{n}{\left(f_{i}\circ f_{i+1}\circ \ldots \circ f_{n}\right)}^{-1}B\left[\left(f_{i}\circ f_{i+1}\circ \ldots \circ f_{n}\right)\left(x\right),\epsilon \right]$ contains no other points of E.*

Modifying slightly the classical Bowen’s definition [18] of the topological entropy of a single map (for details see also Chapter 7 in [21]), we present the definition of topological entropy of solenoids as follows.

**Definition** **2.** *The quantity*

$$h_{top}\left(f_{\infty }\right):=\lim_{\epsilon \to 0^{+}}\underset{n\to \infty }{lim sup}\frac{1}{n}\log s\left(n,\epsilon \right)$$

*is called the topological entropy of the solenoid $f_{\infty }$.*

**Remark** **1.** *The topological entropy of a solenoid can also be expressed in the language of (n,ϵ)-spannings sets. Using arguments similar to remarks on page 169 in [21], we get estimations*

r(n,ϵ)≤s(n,ϵ)≤r(n,ϵ/2).

*Consequently, passing to the suitable limits, we obtain the equality $h_{top}\left(f_{\infty }\right)=\lim_{\epsilon \to 0^{+}}{lim sup}_{n\to \infty }\frac{1}{n}\log r\left(n,\epsilon \right).$*

**Remark** **2.** *Assume that all maps of the sequence $f_{\infty }={\left(f_{n}:X\to X\right)}_{n=1}^{\infty }$ coincide with a fixed continuous map f:X→X of a compact metric space (X,d). Then, the topological entropy of $f_{\infty }$ is equal to the topological entropy of f. For example, the topological entropy of a solenoid coincides with the topological entropy of a nonautonomous dynamical system defined in [11].*

### 2.2. Topological Entropy of a Solenoid via Open Covers

It is a well-known fact that topological entropy of a single continuous map f:X→X can be defined by open covers of the compact metric space (X,d). We intend to show that similar approach can be applied to solenoids. For this purpose, notice that for two open covers A,B of X, the family

A∨B:={A∩B:A∈A,B∈B}

is an open cover of X. Moreover, for a continuous map $f_{i}\circ f_{i+1}\circ \ldots \circ f_{n}:X\to X$ and an open cover A of *X* the family

$${\left(f_{i}\circ f_{i+1}\circ \ldots \circ f_{n}\right)}^{-1}A:=\left\{{\left(f_{i}\circ f_{i+1}\circ \ldots \circ f_{n}\right)}^{-1}A:A\in A\right\}$$

is an open cover of X. Thus, for the open cover A of X, the family

$$\overset{n}{\underset{i=1}{⋁}}{\left(f_{i}\circ f_{i+1}\circ \ldots \circ f_{n}\right)}^{-1}A:=$$

$${\left(f_{1}\circ f_{i+1}\circ \ldots \circ f_{n}\right)}^{-1}\left(A\right)\vee {\left(f_{2}\circ f_{3}\circ \ldots \circ f_{n}\right)}^{-1}\left(A\right)\vee \ldots \vee {\left(f_{n}\right)}^{-1}\left(A\right)$$

is an open cover of X.

For an open cover B of *X* let us denote by N(B) the number of sets in a finite subcover of B covering X, with the smallest cardinality.

**Definition** **3.** *The topological cover entropy of $f_{\infty },$ relative to an open cover A of X, is defined as*

$$h_{top-cov}\left(f_{\infty },A\right):=\underset{n\to \infty }{lim sup}\frac{1}{n}\log N\left(\overset{n}{\underset{i=1}{⋁}}{\left(f_{i}\circ f_{i+1}\circ \ldots \circ f_{n}\right)}^{-1}A\right),$$

*whereas the topological cover entropy of $f_{\infty }$ is the quantity*

$$h_{top-cov}\left(f_{\infty }\right):=\underset{A}{\sup}h\left(f_{\infty },A\right),$$

*where A ranges over all open covers of X.*

### 2.3. Topological Entropy as a Dimension Theory Quantity

Here, we modify the Bowen’s definition [20] of the topological entropy of a continuous single map, which is similar to the construction of the Hausdorff measure, to obtain the topological dimensional entropy of $f_{\infty }$.

#### 2.3.1. The Hausforff Measure and the Hausdorff Dimension

For the convenience of the reader, we recall briefly the classical construction of the Hausdorff measure and the Hausdorff dimension.

For a metric space (X,d) and a subset Y⊂X, let us denote by $Cov_{\epsilon }\left(Y\right)$ the family of open covers B of *Y* with diam(B)<ϵ, for any B∈B. Here, diam(B) denotes the diameter of B.

For any λ>0 the classical Hausdorff λ—measure $\mu_{\lambda }\left(Y\right)$ of a subset Y⊂X is defined as follows,

$$\mu_{\lambda }\left(Y\right):=\lim_{\epsilon \to 0}\inf\left\{\sum_{B\in B}{\left[diam\left(B\right)\right]}^{\lambda }:B\in Cov_{\epsilon }\left(Y\right)\right\}.$$

The function $\lambda \to \mu_{\lambda }\left(Y\right)$ has a unique critical point, where it jumps from *∞* to 0. The Hausdorff dimension HD(Y) of *Y* is defined as the critical point of the function $\lambda \to \mu_{\lambda }\left(Y\right),$ i.e.,

$$HD\left(Y\right)=\sup\left\{\lambda :\mu_{\lambda }\left(Y\right)=\infty \right\}=\inf\left\{\lambda :\mu_{\lambda }\left(Y\right)=0\right\}.$$

#### 2.3.2. Generalized Hausdorff Measure and Generalized Hausdoff Dimension

Arguments similar to the construction of the classical Hausdorff λ-measure and the Hausdorff dimension lead to another entropy-like quantity for $f_{\infty }.$ Denote by A a finite open cover of X. For a subset B⊂X, we write B≺A if there exists $A_{i}\in A$ such that $B\subset A_{i}.$ Denote by $n_{A}\left(B\right)$ the largest non-negative integer *n* such that $f_{1}\circ f_{2}\circ \ldots \circ f_{k}\left(B\right)≺A$ for k=0,1,…,n−1. If there is no element $A_{i}\in A$ such that $B\subset A_{i},$ then we write $n_{A}\left(B\right)=0.$ Let us introduce the following notations.

$$diam_{A}\left(B\right):=exp\left(-n_{A}\left(B\right)\right),$$

$$diam_{A}\left(B\right):=\sup\left\{diam_{A}\left(B\right):B\in B\right\}$$

and

$$D_{A}\left(B,\lambda \right):=\sum_{B\in B}{\left[diam_{A}\left(B\right)\right]}^{\lambda }$$

for a family B of subsets of *X* and a real number, λ>0. For a subset Y⊂X and ϵ>0, let $Cov_{\epsilon }^{A}\left(Y\right)$ denote the family of open covers B of *Y* with $diam_{A}\left(B\right)<\epsilon .$ Now we set

$$\mu_{A,\lambda }\left(Y\right):=\lim_{\epsilon \to 0}\inf\left\{D_{A}\left(B,\lambda \right):B\in Cov_{\epsilon }^{A}\left(Y\right)\right\}.$$

The behavior of the function $\lambda \to \mu_{A,\lambda }\left(Y\right)$ is very similar to the behavior of $\lambda \to \mu_{\lambda }\left(Y\right)$: it has a unique critical point, where it jumps from *∞* to 0. More precisely.

**Lemma** **3.** *For the function $\lambda \to \mu_{A,\lambda }\left(Y\right)$, there exists a unique critical number $\lambda_{0}$ such that $\mu_{A,\lambda }\left(Y\right)=\infty ,\;for\;0\leq \lambda <\lambda_{0}\;and$$\mu_{A,\lambda }\left(Y\right)=0,\;for\;\lambda_{0}<\lambda .$*

**Definition** **4.** *Denote by $\lambda_{0}$ the critical point of the function $\lambda \to \mu_{A,\lambda }\left(Y\right).$ Let $\lambda_{0}=h_{top-dim}\left(\left(f_{\infty }\right),Y,A\right).$ In other words, let*

$$h_{top-dim}\left(\left(f_{\infty }\right),Y,A\right):=\sup\left\{\lambda :\mu_{A,\lambda }\left(Y\right)=\infty \right\}=\inf\left\{\lambda :\mu_{A,\lambda }\left(Y\right)=0\right\}.$$

*The number*

$$h_{top-dim}\left(f_{\infty },Y\right):=\sup\left\{h_{top-dim}\left(\left(f_{\infty }\right),Y,A\right):A\;finite\;open\;cover\;of\;Y\right\}$$

*is called the topological dimensional entropy of $f_{\infty }$ restricted to Y. If Y=X, we write $h_{top-dim}\left(f_{\infty },X\right)=h_{top-dim}\left(f_{\infty }\right).$*

**Remark** **3.** *Our definition of topological dimension entropy of a solenoid is an extension of Bowen’s entropy [20]. Moreover, the topological dimensional entropy of a solenoid is just Bowen’s topological entropy of nonautonomous dynamical systems in [22].*

## 3. Relations between Topological Entropies of a Solenoid

In the previous section, we introduced three entropy-like quantities for a solenoid. Now, we relate the topological dimensional entropy of a solenoid to its topological covering entropy. We obtain the following result.

**Theorem** **1.** $h_{top-dim}\left(f_{\infty }\right)\leq h_{top-cov}\left(f_{\infty }\right).$

**Proof.**  Choose a finite open cover A of *X* and let

$$A_{n}=\left\{\overset{n}{\underset{i=1}{⋂}}{\left(f_{i}\circ f_{i+1}\circ \ldots \circ f_{n}\right)}^{-1}\left(A_{i}\right):A_{i}\in A\right\}.$$

Denote by $B_{n}$ a finite subcover of $A_{n}$ with cardinality $|B_{n}|=N\left(A_{n}\right).$ Then, for any $B\in B_{n},$ we obtain that $n_{A}\left(B\right)\geq n,$ so

$$diam_{A}\left(B\right)\leq \exp\left(-n\right)$$

and for any λ>0 we get

$$D_{A}\left(B_{n},\lambda \right)=\sum_{B\in B_{n}}{\left[diam_{A}\left(B\right)\right]}^{\lambda }=\sum_{B\in B_{n}}exp\left(-\lambda \cdot n_{A}\left(B\right)\right)\leq \left|B_{n}\right|\cdot \exp\left(-\lambda \cdot n\right).$$

As $|B_{n}|=N\left(A_{n}\right),$ we have

$$|B_{n}\left|\cdot \exp\left(-\lambda \cdot n\right)=\exp(-\lambda \cdot n+\log\right|B_{n}\left|\right)=\exp\left(-n\left(\lambda -\frac{1}{n}\log N\left(A_{n}\right)\right)\right).$$

Consequently,

$$D_{A}\left(B_{n},\lambda \right)\leq \exp\left(-n\cdot \left(\lambda -\frac{1}{n}\log N\left(A_{n}\right)\right)\right).$$

Fix ϵ>0 and arbitrary small γ>0. Choose $\lambda^{*}$ such that

$\lambda^{*}>h_{top-cov}\left(f_{\infty },A\right)>\lambda^{*}-\gamma .$ For sufficiently large n∈N, we obtain the inequalities

$$\lambda^{*}-\frac{1}{n}\log N\left(A_{n}\right))>0,$$

$$diam_{A}\left(B\right)<\exp\left(-n\right)<\epsilon ,\;for\;B\in B,and$$

$$D_{A}\left(B_{n},\lambda^{*}\right)\leq \exp\left(-n\cdot \left(\lambda^{*}-\frac{1}{n}\log N\left(A_{n}\right)\right)\right)<\epsilon .$$

As ϵ>0 is arbitrarily small, the above two inequalities yield $\mu_{A,\lambda^{*}}\left(X\right)=0.$ Therefore,

$$h_{top-dim}\left(f_{\infty },Y,A\right)\leq \lambda^{*}\leq h_{top-cov}\left(f_{\infty },A\right)+\gamma .$$

As A is an arbitrary finite open cover of X, we obtain

$$h_{top-dim}\left(f_{\infty }\right)=\sup\left\{h_{top-dim}\left(\left(f_{\infty }\right),X,A\right):A-finite\;open\;cover\;of\;X\right\}$$

$$\leq \sup\left\{h_{top-cov}\left(f_{\infty },A\right):A-finite\;open\;cover\;of\;X\right\}+\gamma =h_{top-cov}\left(f_{\infty }\right)+\gamma .$$

Finally, passing with γ to zero, we get

$$h_{top-dim}\left(f_{\infty }\right)\leq h_{top-cov}\left(f_{\infty }\right).$$

□

**Lemma** **4.** *For an open cover A of X with the Lebegue number Leb(A)=δ, we get*

$$N\left(\overset{n}{\underset{i=1}{⋁}}{\left(f_{i}\circ f_{i+1}\circ \ldots \circ f_{n}\right)}^{-1}A\right)\leq r\left(n,\frac{\delta }{2}\right).$$

**Proof.**  Fix n∈N. Choose an $\left(n,\frac{\delta }{2}\right)$-spanning set *F* with cardinality $card\left(F\right)=r\left(n,\frac{\delta }{2}\right).$ As Leb(A)=δ, we obtain that any ball $B[\left(f_{i}\circ f_{i+1}\circ \ldots \circ f_{n}\right)\left(x\right),\frac{\delta }{2}]$ of radius δ/2, where x∈F and i∈{1,…,n}, is included in some set $A_{i}\in A,$ so

$$\overset{n}{\underset{i=1}{⋂}}{\left(f_{i}\circ f_{i+1}\circ \ldots \circ f_{n}\right)}^{-1}B\left[\left(f_{i}\circ f_{i+1}\circ \ldots \circ f_{n}\right)\left(x\right),\frac{\delta }{2}\right]\subset \overset{n}{\underset{i=1}{⋂}}{\left(f_{i}\circ f_{i+1}\circ \ldots \circ f_{n}\right)}^{-1}A_{i},$$

for some $A_{1},A_{2},\ldots A_{n}\in A.$ It means that the set

$$\overset{n}{\underset{i=1}{⋂}}{\left(f_{i}\circ f_{i+1}\circ \ldots \circ f_{n}\right)}^{-1}B\left[\left(f_{i}\circ f_{i+1}\circ \ldots \circ f_{n}\right)\left(x\right),\frac{\delta }{2}\right]$$

is a subset of some member of the covering

$$\overset{n}{\underset{i=1}{⋁}}{\left(f_{i}\circ f_{i+1}\circ \ldots \circ f_{n}\right)}^{-1}A.$$

On the other hand, applying Lemma 1, we get

$$X=\underset{x\in F}{⋃}\overset{n}{\underset{i=1}{⋂}}{\left(f_{i}\circ f_{i+1}\circ \ldots \circ f_{n}\right)}^{-1}B\left(\left(f_{i}\circ f_{i+1}\circ \ldots \circ f_{n}\right)\left(x\right),\frac{\delta }{2}\right),$$

so

$$N\left(\overset{n}{\underset{i=1}{⋁}}{\left(f_{i}\circ f_{i+1}\circ \ldots \circ f_{n}\right)}^{-1}A\right)\leq card\left(F\right)=r\left(n,\frac{\delta }{2}\right).$$

□

**Lemma** **5.** *Assume that ϵ>0 and B is an open cover of X, with diam(B)≤ϵ. Then,*

$$s\left(n,\epsilon \right)\leq N(\overset{n}{\underset{i=1}{⋁}}{\left(f_{i}\circ f_{i+1}\circ \ldots \circ f_{n}\right)}^{-1}B).$$

**Proof.**  Choose an (n,ϵ)-separated set *E* with cardinality card(E)=s(n,ϵ). Assume that two distinct points $x_{1},x_{2}\in E$ belong to the same member of the cover ${⋁}_{i=1}^{n}{\left(f_{i}\circ f_{i+1}\circ \ldots \circ f_{n}\right)}^{-1}B.$ Therefore, there exist sets $B_{i}\in B$ such that $\left(f_{i}\circ f_{i+1}\circ \ldots \circ f_{n}\right)\left(x_{1}\right),\left(f_{i}\circ f_{i+1}\circ \ldots \circ f_{n}\right)\left(x_{2}\right)\in B_{i}$ for any i∈{1,…,n}. On the other hand, as the set *E* is (n,ϵ)-separated, there exists j∈{1,…,n} such that

$$d(\left(f_{j}\circ f_{j+1}\circ \ldots \circ f_{n}\right)\left(x_{1}\right),\left(f_{j}\circ f_{j+1}\circ \ldots \circ f_{n}\right)\left(x_{2}\right))=$$

$$\max\{d\left(f_{i}\circ f_{i+1}\circ \ldots \circ f_{n}\right)\left(x_{1}\right),\left(f_{i}\circ f_{i+1}\circ \ldots \circ f_{n}\right)\left(x_{2}\right):i\in \left\{1,\ldots ,n\right\}\}>\epsilon .$$

Thus, we get a contradiction with $diam(B_{j})\leq \epsilon .$ Therefore,

$$s\left(n,\epsilon \right)\leq N(\overset{n}{\underset{i=1}{⋁}}{\left(f_{i}\circ f_{i+1}\circ \ldots \circ f_{n}\right)}^{-1}B).$$

□

Now, we are ready to prove that the topological entropy of a solenoid is equivalent to its topological covering entropy.

**Theorem** **2.** $h_{top}\left(f_{\infty }\right)=h_{top-cov}\left(f_{\infty }\right).$

**Proof.**  Fix ϵ>0. Let $A_{\epsilon }$ be the cover of *X* by all open balls of radius 2·ϵ and denote by $B_{\epsilon }$ the cover of *X* by all open balls of radius $\frac{\epsilon }{2}.$ Due to Lemma 4, we obtain

$$N\left(\overset{n}{\underset{i=1}{⋁}}{\left(f_{i}\circ f_{i+1}\circ \ldots \circ f_{n}\right)}^{-1}B\right)\leq r\left(n,\epsilon \right),$$

so

$$\underset{n\to \infty }{lim sup}\frac{1}{n}\log N\left(\overset{n}{\underset{i=1}{⋁}}{\left(f_{i}\circ f_{i+1}\circ \ldots \circ f_{n}\right)}^{-1}A_{\epsilon }\right)\leq \underset{n\to \infty }{lim sup}\frac{1}{n}\log r\left(n,\epsilon \right)$$

and

$$h_{top-cov}\left(f_{\infty }\right)\leq h_{top}\left(f_{\infty }\right).$$

Applying Lemma 5, we get

$$s\left(n,\epsilon \right)\leq N(\overset{n}{\underset{i=1}{⋁}}{\left(f_{i}\circ f_{i+1}\circ \ldots \circ f_{n}\right)}^{-1}B_{\epsilon },$$

so

$$\underset{n\to \infty }{lim sup}\frac{1}{n}\log s\left(n,\epsilon \right)\leq \underset{n\to \infty }{lim sup}\frac{1}{n}\log N(\overset{n}{\underset{i=1}{⋁}}{\left(f_{i}\circ f_{i+1}\circ \ldots \circ f_{n}\right)}^{-1}B_{\epsilon }$$

and finally we get the second inequality

$$h_{top}\left(f_{\infty }\right)\leq h_{top-cov}\left(f_{\infty }\right).$$

The theorem is proved. □

## 4. Topological Entropy of L-Lipschitz Solenoids

Dai, Zhou, and Geng [23] proved the following result. If X is a metric compact space and f:X→X a Lipschitz continuous map, then the Hausdorff dimension of X is lower estimated by the topological entropy of f divided by the logarithm of its Lipschitz constant. In 2004, Misiurewicz [24] provided a new definition of topological entropy of a single transformation, which was a kind of hybrid between the Bowen’s definition and the original definition of Adler, Konheim, and McAndrew [17]. The main theorem in [24] is similar to the result in [23]. In this section, we consider a special class of solenoids called *L-Lipschitz solenoids*. We say that a solenoid determined by $f_{\infty }={\left(f_{n}:X\to X\right)}_{n=1}^{\infty }$ is a *L-Lipschitz* if there exists L>0 such that each map $f_{n}:X\to X$ is an Lipschitz epimorphism with Lipschitz constant *L*, i.e., for any x,y∈X and arbitrary n∈N

$$d(f_{n}\left(x\right),f_{n}\left(y\right))\leq L\cdot d\left(x,y\right).$$

Let us start with the following example.

**Example** **1.** *Consider the solenoid $f_{\infty }={\left(f_{n}:T^{2}\to T^{2}\right)}_{n=1}^{\infty },$ where $T^{2}=\frac{R^{2}}{Z^{2}}$ is two-dimensional torus and each $f_{n}:T^{2}\to T^{2}$ is the doubling map, i.e., $f_{n}\left(x_{1},x_{2}\right)=2\cdot \left(x_{1},x_{2}\right),$ for any $\left(x_{1},x_{2}\right)\in T^{2}$. Then,*

$$\frac{h_{top}\left(f_{\infty },T^{2}\right)}{\log(2)}=HD\left(T^{2}\right)=\frac{h_{top-dim}\left(f_{\infty },T^{2}\right)}{\log(2)}.$$

Indeed, the Hausdorff dimension of the two dimensional torus is equal to two (see page 23 in [25]). Due to Remarks 2 and 3, we get $h_{top}\left(f_{\infty },T^{2}\right)=h_{top}\left(f_{2}\right)=h_{top-dim}\left(f_{\infty },T^{2}\right).$ On the other hand, the doubling map $f_{2}:T^{2}\to T^{2}$ can be considered as the Cartesian product of two doubling maps $g:\frac{R}{Z}\to \frac{R}{Z}$ defined by g(x)=2·xmod1, for $x\in \frac{R}{Z}.$ Moreover, $h_{top}\left(g\right)=\log\left(2\right)$ (see Example on page 29 in [26]). Consequently, $h_{top}\left(f_{\infty },T^{2}\right)=2\cdot \log\left(2\right)=h_{top-dim}\left(f_{\infty },T^{2}\right).$

To show the comprehensive picture of dynamics of L-Lipschitz solenoids, we rewrite the Theorem 3 published in [11], written for nonautonomous dynamical systems, in the set up of solenoids as follows.

**Theorem** **3.** *Assume that $f_{\infty }={\left(f_{n}:X\to X\right)}_{n=1}^{\infty }$ is a L-Lipschitz solenoid with L>1. Then, for any Y⊂X, we obtain*

$$HD\left(Y\right)\geq \frac{h_{top-dim}\left(\left(f_{\infty }\right),Y\right)}{\log(L)}.$$

For the convenience of the reader and to make the paper self-contained, we write the proof of Theorem 3 which is essentially the same as the proof of Theorem 3 in [11].

**Proof.**  Choose a finite open cover A of *Y* and denote by δ=Leb(A) its Lebesgue number. It means that for an open subset C⊂Y with diameter diam(C)<δ, there exists A∈A such that C⊂A. Choose an open set *B* with $\frac{\delta }{L^{n}}<diam\left(B\right)<\frac{\delta }{L^{n-1}}.$ We obtain that

$$diam(f_{1}\circ f_{2}\circ \ldots .\circ f_{k}\left(B\right))<\delta$$

for any k=1,2,…n−1, so $n_{A}\left(B\right)\geq n$. From the inequality

$$\frac{\delta }{L^{n}}<diam\left(B\right)$$

we conclude that

$$n>\frac{\log(\delta )-\log(diam(B))}{\log(L)}.$$

Consequently,

$$\frac{\log(\delta )-\log(diam(B))}{\log(L)}\leq n_{A}\left(B\right)$$

and

$$diam_{A}\left(B\right)=\exp\left(-n_{A}\left(B\right)\right)\leq \exp\left(-\frac{\log(\delta )-\log(diam(B))}{\log(L)}\right)=$$

$$\exp\left[-\left(\frac{\log(\delta )}{\log(L)}\right)\right]\cdot {\left(diam\left(B\right)\right)}^{\frac{1}{\log(L)}}.$$

Therefore, for an open cover B of *Y* consisting of open sets *B* with $\frac{\delta }{L^{n}}<diam\left(B\right)<\frac{\delta }{L^{n-1}}$ and λ>0, we get

$$D_{A}\left(B,\lambda \right)\leq \exp\left[-\lambda \cdot \left(\frac{\log(\delta )}{\log(L)}\right)\right]\cdot \sum_{B\in B}(diam{\left(B\right)}^{\frac{\lambda }{\log(L)}}.$$

Fix γ>0 and choose $\lambda_{1}$ such that

$$\frac{\lambda_{1}}{\log(L)}>HD\left(Y\right)\geq \frac{\lambda_{1}}{\log(L)}-\gamma .$$

By definition of the Hausdorff measure, the equality $\mu_{\frac{\lambda_{1}}{\log(L)}}\left(Y\right)=0$ holds. Therefore, for any ϵ>0 there exists and an open cover $B_{\epsilon }$ of *Y* such that for any $B\in B_{\epsilon }$

$$\epsilon >\exp\left[-\left(\frac{\log(\delta )}{\log(L)}\right)\right]\cdot (diam{\left(B\right)}^{\frac{1}{\log(L)}}>diam_{A}\left(B\right)$$

and

$$\epsilon >\exp\left[-\lambda_{1}\cdot \left(\frac{\log(\delta )}{\log(L)}\right)\right]\cdot \sum_{B\in B_{\epsilon }}(diam{\left(B\right)}^{\frac{\lambda_{1}}{\log(L)}}>D_{A}\left(B_{\epsilon },\lambda_{1}\right).$$

The inequalities

$$\mu_{A,\lambda_{1}}\left(Y\right)\leq D_{A}\left(B_{\epsilon },\lambda_{1}\right)<\epsilon$$

yield $\mu_{A,\lambda_{1}}\left(Y\right)=0.$ According to Definition 4, we get

$$h_{top-dim}\left(\left(f_{\infty }\right),Y,A\right)=\inf\left\{\lambda :\mu_{A,\lambda }\left(Y\right)=0\right\}\leq \lambda_{1}.$$

Taking supremum over all open finite covers of Y, we obtain

$$h_{top-dim}\left(\left(f_{\infty }\right),Y\right)=$$

$$\sup\{h_{top-dim}\left(\left(f_{\infty }\right),Y,A\right):A-finite\;open\;cover\;of\;Y\}\leq$$

$$\lambda_{1}\leq \log\left(L\right)\cdot \left(HD\left(Y\right)+\gamma \right).$$

Finally,

$$h_{top-dim}\left(\left(f_{\infty }\right),Y\right)\leq \log\left(L\right)\cdot HD\left(Y\right),$$

as γ is an arbitrarily small positive number. □

In particular, taking Y=X, we obtain the following corollary.

**Corollary** **1.** *Assume that $f_{\infty }={\left(f_{n}:X\to X\right)}_{n=1}^{\infty }$ is a L-Lipschitz solenoid. Then, the inequality*

$$HD\left(X\right)\geq \frac{h_{top-dim}\left(\left(f_{\infty }\right)\right)}{\log(L)}$$

*holds.*

In the special case, for $f_{\infty }={\left(f_{n}:X\to X\right)}_{n=1}^{\infty }$ being a L-Lipschitz solenoid such that all maps $f_{n}:X\to X$ coincide with a continuous map f:X→X, we get that

$$h_{top}\left(f_{\infty }\right)=h_{top}\left(f_{2}\right),$$

where $h_{top}\left(f_{2}\right)$ is the classical topological entropy of $f_{2}:X\to X.$ Bowen proved (Proposition 1 in [20]) that $h_{top-dim}\left(f_{2}\right)=h_{top}\left(f_{2}\right).$ Consequently, as a corollary of Theorem 3, we get the result of Misiurewicz [24].

**Corollary** **2** (Theorem 2.1 in [24])**.** *If f:X→X is a continuous L-Lipschitz of a compact metric space (X,d), then*

$$HD\left(X\right)\geq \frac{h_{top}\left(f\right)}{\log(L)}.$$

## 5. Topological Entropy of Locally Expanding Solenoids

In this section, we investigate the dynamics of locally expanding solenoids. Ruelle [16] introduced the notion of a locally expanding map in the following way.

**Definition** **5.** *Let (X,d) be a compact metric space and f:X→X a continuous selfmap. If for λ>1 there exists ϵ>0 such that for every pair of distinct points x,y∈X*

d(x,y)<ϵ⇒d(f(x),f(y))≥λ·d(x,y),

*then we say that f is a locally (ϵ,λ)-expanding map and λ is an* expanding coefficient *of f.*

Notice that any finite composition of locally $\left(\epsilon_{i},\lambda \right)$-expanding maps is an (ϵ,λ)-locally expanding map for some ϵ>0. We extend the notion of locally expanding map to a solenoid as follows.

**Definition** **6.** *Given a solenoid $f_{\infty }={\left(f_{n}:X\to X\right)}_{n=1}^{\infty }$, if all maps $f_{n}:X\to X$ are locally $\left(\epsilon_{n},\lambda \right)$-expanding, then we say that the solenoid $f_{\infty }$ is locally λ-expanding.*

**Lemma** **6.** *Given a locally λ-expanding solenoid determined by a sequence $f_{\infty }={\left(f_{n}:X\to X\right)}_{n=1}^{\infty }$. Then, for any k∈N, there exists $\delta_{k}>0$ such that for every pair of distinct points x,y∈X*

$$d\left(x,y\right)<\delta_{k}\Rightarrow \;d\left(f_{1}\circ f_{2}\circ \ldots \circ f_{k}\left(x\right),f_{1}\circ f_{2}\circ \ldots \circ f_{k}\left(y\right)\right)\geq \lambda^{k}\cdot d\left(x,y\right).$$

*Moreover, for any $\gamma \in (0,\delta_{k})$,*

$$B_{k}^{f_{\infty }}\left(x,\gamma \right):=\overset{k}{\underset{i=1}{⋂}}{\left(f_{1}\circ f_{2}\circ \ldots \circ f_{k}\right)}^{-1}B\left(f_{1}\circ f_{2}\circ \ldots \circ f_{k}\left(x\right),\gamma \right)\subset B\left(x,\frac{\gamma }{\lambda^{k}}\right).$$

**Proof.**  A composition of *k* locally expanding maps is again a locally expanding map. Therefore, there exists $\delta_{k}>0$ such that for every pair of distinct points x,y∈X, we get

$$d\left(x,y\right)<\delta_{k}\Rightarrow \;d\left(f_{1}\circ f_{2}\circ \ldots \circ f_{k}\left(x\right),f_{1}\circ f_{2}\circ \ldots \circ f_{k}\left(y\right)\right)\geq$$

$$\lambda \cdot d(f_{2}\circ \ldots \circ f_{k}\left(x\right),f_{2}\circ \ldots \circ f_{k}\left(y\right))\geq \lambda \cdot \ldots \cdot \lambda \cdot d\left(x,y\right)=$$

$$\lambda^{k}\cdot d\left(x,y\right).$$

Fix $\gamma \in (0,\delta_{k})$ and choose $y\in B_{k}^{f_{\infty }}\left(x,\gamma \right)$, then

$$d(\left(f_{1}\circ f_{2}\circ \ldots \circ f_{i}\right)\left(x\right),\left(f_{1}\circ f_{2}\circ \ldots \circ f_{i}\right)\left(y\right))<\gamma$$

for every i∈{1,2,…,k}. If y=x, then clearly $x\in B\left(x,\frac{\gamma }{\lambda^{k}}\right)$. Therefore, assume that y≠x, as $d\left(x,y\right)<\gamma <\delta_{k}$, we get inequalities

$$\gamma >d(f_{1}\circ f_{2}\circ \ldots \circ f_{k}\left(x\right),f_{1}\circ f_{2}\circ \ldots \circ f_{k}\left(y\right))\geq$$

$$\lambda \cdot d\left(f_{2}\circ \ldots \circ f_{k}\left(x\right),f_{2}\circ \ldots \circ f_{k}\left(y\right)\right)\geq \lambda^{k}d\left(x,y\right).$$

Therefore, $d\left(x,y\right)<\frac{\gamma }{\lambda^{k}}$ and $y\in B(x,\frac{\gamma }{\lambda^{k}}).$ The lemma is proved. □

The notion of the box dimension is an example of fractal dimension which belongs to fractal geometry. It was defined independently by Minkowski and Bouligard for a subset of Euclidean space. For modern presentation of fractal dimensions see the classical books of Falconer [25,27] or the monograph written by Przytycki and Urbański [28].

**Definition** **7** (Chapter 2 in [25])**.** *Recall that the upper box dimension of a closed subset Z of a compact metric space X is*

$$\bar{\dim_{B}\left(Z\right)}:=\underset{\gamma \to 0}{lim sup}\frac{\log N(Z,\gamma )}{-\log\gamma },$$

*where N(Z,γ) denotes the smallest number of balls B(x,γ) of radius γ>0 needed to cover Z.*

**Lemma** **7** ([28])**.** *For a compact metric space X, the Hausdorff dimension HD(X) of X and the upper box dimension $\bar{\dim_{B}\left(X\right)}$ of X are interrelated*

$$HD\left(X\right)\leq \bar{\dim_{B}\left(X\right)}.$$

In the proof of Theorem 4 we need the following lemma.

**Lemma** **8** (Lemma 6.2 in [29])**.** *Let $\phi :R\to R_{+}$ be a decreasing function. If δ∈(0,1) and γ>0, then*

$$\underset{r\to 0}{lim sup}\frac{\log\phi (r)}{\log r}=\underset{n\to \infty }{lim sup}\frac{\log\phi (\delta^{n}\gamma )}{\log(\delta^{n}\gamma )}.$$

**Theorem** **4.** *Given a locally λ-expanding solenoid $f_{\infty }={\left(f_{n}:X\to X\right)}_{n=1}^{\infty }$. Then,*

$$h_{top}\left(f_{\infty }\right)\geq \left(\log\lambda \right)\cdot \bar{\dim_{B}\left(X\right)}\geq \left(\log\lambda \right)\cdot HD\left(X\right).$$

**Proof.**  In the first part of the proof we intend to show that

(1) $$h_{top}\left(f_{\infty }\right)\geq \left(\log\lambda \right)\cdot \bar{\dim_{B}\left(X\right)}.$$

Let ϵ>0 and λ>1 be such that for every pair of distinct points x,y∈X and for every i∈{1,…,k},

$$d\left(x,y\right)<\epsilon \Rightarrow d(f_{i}\left(x\right),f_{i}\left(y\right))\geq \lambda \cdot d\left(x,y\right).$$

By Lemma 6, for γ∈(0,ϵ) and n∈N, we have

(2) $$N\left(X,\frac{\gamma }{\lambda^{n}}\right)\leq r\left(n,\gamma \right),$$

consequently, applying Lemma 8 for the first equality and (2) for the subsequent inequality, we get

$$\bar{\dim_{B}\left(X\right)}=\underset{n\to \infty }{lim sup}\frac{\log N(X,\frac{\gamma }{\lambda^{n}})}{-\log\frac{\gamma }{\lambda^{n}}}\leq \underset{n\to \infty }{lim sup}\frac{\log r(n,\gamma )}{-\log\frac{r}{\lambda^{n}}}=$$

$$\frac{1}{\log\lambda }\cdot \underset{n\to \infty }{lim sup}\frac{\log r(n,\gamma )}{n}.$$

Therefore,

$$h_{top}\left(f_{\infty }\right)=\lim_{\gamma \to 0}\underset{n\to \infty }{lim sup}\frac{\log r(n,\gamma )}{n}\geq \left(\log\lambda \right)\cdot \bar{\dim_{B}\left(X\right)}.$$

According to the Lemma 6, we finally get

$$h_{top}\left(f_{\infty }\right)\geq \left(\log\lambda \right)\cdot \bar{\dim_{B}\left(X\right)}\geq \left(\log\lambda \right)\cdot HD\left(X\right).$$

□
