# Correction: Biś, A., et al. Hausdorff Dimension and Topological Entropies of a Solenoid. *Entropy* 2020, *22*, 506

**DOI:** 10.3390/e22101172

**Published:** 2020-10-19

**Authors:** Andrzej Biś, Agnieszka Namiecińska

**Affiliations:** Faculty of Mathematics and Computer Science, Banacha 22, 90-235 Łódź, Poland; a.namiecinska@wp.pl

The authors wish to make the following corrections to this paper [1]:

On page 1, the paper erroneously states that the solenoid *X*_∞_ is uniquely determined by the sequence of epimorphisms *f*_∞_. Consequently, the results of the paper do not apply to solenoids, understood as geometric objects that arose due to an inverse limit construction. The results of the paper apply only to the dynamics of the sequences of epimorphisms, for which their inverse limits are well defined.

The authors would like to apologize for any inconvenience caused to the readers by these changes.
